# Dimensional analysis of MINMOD leads to definition of the disposition index of glucose regulation and improved simulation algorithm

**DOI:** 10.1186/1475-925X-5-44

**Published:** 2006-07-14

**Authors:** Aparna Nittala, Soumitra Ghosh, Darko Stefanovski, Richard Bergman, Xujing Wang

**Affiliations:** 1 Max McGee National Research Center for Juvenile Diabetes & Human and Molecular Genetics Center, Medical College of Wisconsin and Children's Research Institute of the Children's Hospital of Wisconsin, 8701 Watertown Plank Road, Milwaukee, Wl 53226, USA; 2 Department of Physiology & Biophysics, Medicine, Biomedical Engineering Keck School of Medicine, Viterbi School of Engineering, University of Southern California, 1975 Zonal Avenue, Los Angeles, California 90089-9023, USA

## Abstract

**Background:**

Frequently Sampled Intravenous Glucose Tolerance Test (FSIVGTT) together with its mathematical model, the minimal model (MINMOD), have become important clinical tools to evaluate the metabolic control of glucose in humans. Dimensional analysis of the model is up to now not available.

**Methods:**

A formal dimensional analysis of MINMOD was carried out and the degree of freedom of MINMOD was examined. Through re-expressing all state variable and parameters in terms of their reference scales, MINMOD was transformed into a dimensionless format. Previously defined physiological indices including insulin sensitivity, glucose effectiveness, and first and second phase insulin responses were re-examined in this new formulation. Further, the parameter estimation from FSIVGTT was implemented using both the dimensional and the dimensionless formulations of MINMOD, and the performances were compared utilizing Monte Carlo simulation as well as real human FSIVGTT data.

**Results:**

The degree of freedom (DOF) of MINMOD was found to be 7. The model was maximally simplified in the dimensionless formulation that normalizes the variation in glucose and insulin during FSIVGTT. In the new formulation, the disposition index (Dl), a composite parameter known to be important in diabetes pathology, was naturally defined as one of the dimensionless parameters in the system. The numerical simulation using the dimensionless formulation led to a 1.5–5 fold gain in speed, and significantly improved accuracy and robustness in parameter estimation compared to the dimensional implementation.

**Conclusion:**

Dimensional analysis of MINMOD led to simplification of the model, direct identification of the important composite factors in the dynamics of glucose metabolic control, and better simulations algorithms.

## Background

To mathematically model a physiological mechanism, the factors that govern its *modus operandi *can be taken as independent or dependent variables in the mathematical model and the behavior of the whole system be described by a set of simple or complex, differential or integral equations. The minimal model of glucose regulation, MINMOD, is one such model. First formulated and introduced by Richard Bergman and colleagues, it describes the kinetics of plasma glucose and insulin during a Frequently Sampled Intravenous Glucose Tolerance Test (FSIVGTT), and allows dissection of the composite effects of insulin secretion and insulin sensitivity on glucose tolerance and risk for diabetes [1-3]. The model was termed as "minimal" for it was the least complex mathematical representation that is capable of accounting for the observed dynamic relationship between insulin and glucose disappearance [1,2]. In FSIVGTT, after overnight fasting, individual subjects are given an initial infusion of glucose bolus of 300 mg/kg body weight at the beginning of the experiments. At periodic time intervals afterwards, blood samples for glucose and insulin measurement will be taken for up to 180 minutes -normally, every 2–5 minutes within the first 30 minutes, every 5–10 minutes for 30–60 minutes, and every 30 minutes from 60 to 180 minutes. Mathematically, the regulation of plasma glucose concentration *G*(*t*) was formulated to be [1-3]:



where *G*_*b *_is the baseline glucose concentration, *p*_*1 *_is the insulin-independent glucose disappearance rate, and *X*(*t*) is an auxiliary function representing insulin-excitable tissue glucose uptake activity. It is proportional to the insulin concentration in a "remote" interstitial compartment (which was later shown to be the interstitial fluid [4]), and is described by



where *l*_*b *_is the baseline insulin concentration, *p*_*2 *_is the rate constant of the spontaneous decrease in *X*(*t*), and *p*_*3 *_is the rate of insulin-dependent increase in tissue *X*(*t*). The change in insulin is given by



where *n *is the disappearance rate of endogenous insulin, *γ*(*G *- *h*) *t *is the Insulin Delivery Rate (IDR) of second phase insulin secreted into the venous circulation (corrected for hepatic extraction), and *h *is the threshold value of glucose above which the endogenous insulin secretion will be stimulated. Under stimulatory glucose exposure, insulin release from pancreas normally is biphasic. More details of the parameters used in MINMOD can be found in table 1B. The initial conditions are assumed to be: *G*(0) = *G*_0_, *X*(0) = 0, and *I*(0) = *I*_0_, and the equilibrium state is given by *G*(∞) = *G*_*b*_, *X*(∞) = 0, and *I*(∞) = *I*_*b*_. Using this model, parameter values can be estimated from FSIVGTT measurements, and four independent parameters can be derived that were believed to represent a comprehensive metabolic portrait of an individual [1,2,5]:

**Table 1B:** Parameters in MINMOD.

**Quantity**	**Description**	**Value and Unit**	**Dimensions**		
			
			**M**	**L**	**T**
*p*_1_	Insulin-independent glucose disappearance rate. Also known as glucose effectiveness (S_G_).	~10^-2 ^min^-1^	0	0	-1
*p*_2_	Rate constant expressing the spontaneous decrease of tissue glucose uptake ability.	~10^-2 ^min^-1^	0	0	-1
*p*_3_	Insulin-dependent increase in tissue glucose uptake ability, per unit of insulin concentration excess over baseline insulin.	~10^-5 ^min^-2 ^(μU/ml)^-1^	-1	3	-2
*n*	Disappearance rate of endogenous insulin.	~10^-1 ^min^-1^	0	0	-1
*γ*	Rate of second phase endogenous insulin secretion	~10^-2^–10^-3 ^(μU/ml) min^-2^	0	0	-2
*G*_*b*_	Baseline plasma glucose	~100 mg/dl	1	-3	0
*G*_0_	Initial glucose concentration during FSIVGTT	~300 mg/dl	1	-3	0
*h*	A threshold value (higher than basal) for plasma glucose above which the second phase insulin secretion is stimulated	~100 mg/dl	1	-3	0
*l*_*b*_	Baseline plasma insulin	~10 μU/ml	1	-3	0
*l*_0_	Initial insulin concentration	~30 μU/ml	1	-3	0

• Insulin sensitivity: , where cap dot represents time derivative, and SS stands for steady state. This is a measure of the capability of insulin-stimulated glucose uptake.

• Glucose effectiveness: . It measures the glucose facilitated, insulin-independent glucose uptake.

• First phase pancreatic responsiveness , where *AIR *= (*I*_0 _- *I*_*b*_)/*n *(Acute Insulin Response) is the total insulin release during first phase [6], and *φ*_1 _measures first phase insulin release per unit rise of glucose above basal.

• Second phase pancreatic responsiveness *φ*_2 _= *∂*^2 ^(*IDR*_2_)/*∂G∂t *= *γ*, is the dependence of rate of rise of the 2^nd ^phase insulin secretion on glucose.

*S*_*I *_later led to the definition of the disposition index DI = *S*_I _* AIR [7,8] (notice that it is dimensionless). Bogardus and colleagues have demonstrated that Dl was an excellent predictor (prognostic index) for which individual will develop type 2 diabetes mellitus (T2DM) in the Pima native American population [9]. In addition, *S*_*I *_was found to possess greater heritability than indices defined by other models including the homeostasis or the fasting insulin model assessments [4,10]. The model is now the basis for a large number of laboratory and clinical investigations (~50 reports/year) [4,11]. According to the American Diabetes Association Consensus Development Conference on insulin resistance [12], it is one of the only two methods (the other one is the euglycemic insulin clamp) that are recommended for assessing peripheral insulin resistance due to their satisfactory, consistent performance.

Since the publication of MINMOD, its mathematical structure, system properties, as well as simulation techniques of its computer program have also been studied [11,13,14]. However, up to now, a dimensional analysis is still lacking. How many degrees of freedom (DOF) does a system described by MINMOD have? How many independent indices can be defined from the model to characterize the system? How many indices are needed to differentiate different pathological states of glucose metabolic control? These questions have not been examined in depth. In addition, in a mathematical model, each variable or parameter has an associated dimension or unit that reflects its influence on the system behavior. One could carry out model analysis and computer simulation by taking the parameters as they are, with their respective units, as has been done with MINMOD up till now. There are certain disadvantages with such approaches. The parameters do not necessarily have the same range of values. While some vary within a narrow scale, others may span a wide range such that the absolute values of these parameters can be extremely different from each other. This makes it difficult to compare the relative importance of the parameters in controlling the system properties, as there is no uniform scale – a reference scale – based on which all the parameters can be studied [15].

Dimensional analysis is a process to simplify mathematical models expressed in differential equations [16,17]. The technique rescales every variable and parameters in terms of their intrinsic reference quantities so that the equations can be expressed in terms of dimensionless variables and parameters whose typical scales are all ~O(1) [16]. An intrinsic reference quantity is one that reflects the intrinsic value scale of the variable; it can be its basal or maximal value, or its dynamic range of variation, for example. This process reduces the number of variables by removing redundant degrees of freedom. Using it, one can analyze the behavior of the system regardless of the units used to measure the variables. The dimensionless formulation helps to identify the dominant terms in the equations, as well as their interactions in the model behavior and their influence on the solution structure. It reveals which variables, or rates of change, can be thought of as small, or even 'negligible' relative to others. In addition, the process often reveals that some of the parameters do not affect the system's dynamic behavior independently, and they can be combined into dimensionless indices that reflect their collective effect. Such indices are usually the most effective predictors of system behavior. Examples include the Reynolds number (the ratio of the inertial force to the viscous force) in fluidics, the R number in epidemiology of infections, and the ratio of tumor growth to normal growth in oncology. Furthermore, with dimensional analysis, one can rescale models to duplicate the behavior of the original system provided that the governing dimensionless parameters have the same values in the two systems [18]. This will allow the identification of scale-invariant parameters, and the translation between animal models and human studies.

In this paper we carry out dimensional analysis of MINMOD. We show that it leads to the direct identification of important pathological indices. Further, we implement the computer simulation of MINMOD using the dimensionless formulation of glucose regulation. Utilizing Monte Carlo simulation as well as real human FSIVGTT data, we compare the new implementation to the original dimensional implementation in terms of model fit, speed of convergence, accuracy of parameter estimation and robustness against noise.

## Methods

### Dimensional analysis of MINMOD

#### The dimensions of the state variables and parameters

The mathematical structure of MINMOD consists of two first-order ordinary differential equations (ODE) for glucose disappearance (equations 1A-B) and one first-order ODE for insulin kinetics (equation 1C). The state variables and parameters along with their normal value ranges and units, as well as their dimensions in terms of the fundamental units of mass (M), length (L) and time (T) are presented in table 1A and 1B.

**Table 1A:** State variables, their meanings, values ranges in humans, units, and dimensions in terms of the fundamental units of mass (M), length (L) and time (T).

**Quantity**	**Description**	**Value and Unit**	**Dimension**		
			
			**M**	**L**	**T**
*G*(*t*)	Plasma glucose concentration at time t	~100–300 mg/dl	1	-3	0
*l*(*t*)	Plasma insulin concentration at time t	~10–30 μU/ml	1	-3	0
*X*(*t*)	An auxiliary function proportional to insulin concentration in the interstitial compartment.	~0–10^-3^min^-1^	0	0	-1

There are totally 10 parameters (*p*_*1*_, *p*_*2*_, *p*_*3*_, *n*, *γ*, *h*, *G*_*b*_, *G*_*0*_, *l*_*b*_, *l*_*0*_) in MINMOD, expressed in a total of 3 fundamental units. According to Buckingham's Pi theorem [17,19], the DOF of this system is 7, implying that the model can be described in a simplified form with 7 free dimensionless parameters. Usually there is more than one way to non-dimensionalize a multi-parameter model, and it is worthwhile to make a choice that is meaningful and also maximally simplifies the equations. For example, it would be worthwhile to carefully define the intrinsic reference scale for each quantity, such that all rescaled dimensionless quantities vary within the same order of magnitude in value.

Among the three state variables, only *G*(*t*) and *l*(*t*) can be measured. *X*(*t*), which reflects the amount of insulin in the interstitial compartment, cannot be measured directly. In addition, equation 1B can be solved with . In FSIVGTT *X*(0) = 0, it follows that . Therefore we will focus on the reference choices for *G *and *l*, and explore and compare several means of non-dimensionalization of MINMOD.

#### Non-dimensionalization choice a, rescale time by the glucose disappearance rate

One natural choice to rescale *G *and *l *is with their baseline values and define . In the model fitting of FSIVGTT with MINMOD, we usually start with the second time point when the plasma glucose and insulin concentrations have peaked after the initial glucose bolus. Therefore the initial values of *G*_0 _and *I*_0 _are also the maxima for the glucose and insulin secretion (during the second phase of endogenous insulin release, the insulin concentration can peak again, but normally with a much lower peak value than in the first phase release). Under this choice, the rescaled glucose and insulin concentration vary between  and , respectively. It might be argued that in the effort to uptake the exogenous glucose, the system can overcorrect and reach a concentration lower than *G*_*b *_before it reaches the equilibrium value of *G*_*b*_. De Gaetano et al have carried out a steady state analysis of MINMOD, and showed that likely the system approaches the steady state *G*_*b *_from below [13]. Even if this is the case, the minimal value is still very close to *G*_*b*_, with .

There are two natural time scales in this system, 1/*p*_1 _the time scale of the glucose disappearance on its own, and 1/*n *the time scale of the insulin disappearance. If we chose to use 1/*p*_1 _to rescale time, equations 1A–1C become:



with *τ *= *p*_1_*t*, , and . The initial conditions become: , and . All the barred variables and parameters are unitless. The number of free parameters in the system indeed reduces to 7: , and [_0_].

#### Choice b, rescale time by the insulin disappearance rate

Similarly, we can rescale time by 1/*n*, the disappearance rate of endogenous insulin. In this choice the dimensionless parameters are *τ *= *nt*, , and , and the differential equations become



With initial conditions: (0) = _0_, and (0) = _0_. The number of free parameters again reduces to 7 as expected: , and _0_.

#### Choice c, rescale to normalize the variation in glucose and insulin

A more sophisticated choice is to rescale glucose and insulin in a way to ensure that they vary within the same scale, by defining . Now  ∈ [0,1], and  ∈ [0,1]. Normally insulin facilitated glucose disappearance is the major mode of glucose uptake, and insulin's own kinetics will directly impact on that. Therefore we chose to rescale time by the disappearance rate of endogenous insulin during first phase secretion: *τ *= *nt*. It follows that other parameters can be defined by: , and . The equations become:



where , and . The initial conditions become: (0) = 1, and (0) = 1 and equilibrium state has (∞) = 0, and (∞) = 0. Therefore both glucose and insulin vary within a unit range of [0, 1]. The 7 free parameters are: , and _1_. Among the three choices, choice c maximally simplifies the equations and the initial conditions.

### Computer simulation of glucose disappearance in the dimensionless formulation

A computer program to simulate the glucose disappearance component of MINMOD is developed by us in Matlab using both the dimensional and the dimensionless formulations. For dimensionless implementation, the formulation in choice c was adopted. The minimization function Isqnonlin.m available in the optimization toolbox of Matlab was utilized for parameter estimation. The program flow is similar as described in figure 1 of [3], and the same weighting scheme of the measured data points is adopted. In the initial computer implementation of MINMOD reported in [3], both glucose and insulin profiles were fitted by the mathematical model (equations 1A–1C). FSIVGTT and MINMOD were first developed utilizing a dog model. Later, it was found that as humans are more insulin resistant [8], and more endogenous insulin secretion is needed to generate sufficient insulin action in order for the computer program to accurately estimate the model parameters. It was found that this demand was more imperative for assessment of insulin sensitivity in diabetic (both type 1 and type 2 diabetes) subjects, as these individuals exhibit diminished or absent insulin-secretory response and/or higher insulin resistance. To overcome this problem, modified FSIVGTT protocol was introduced where a second injection of 100–300 mg tolbutamide [20] or 30 mU/kg body weight insulin [21] was infused 20 min after the initial glucose bolus. In these protocols, while the glucose regulation can still be adequately described by equations 1A–1B, the insulin regulation will require modifications of the original model given in equation 1C. In view of the variations in FSIVGTT protocols presently used in clinical practice, later computer simulations of MINMOD mainly focused on the glucose regulation, and the estimation of parameters *p*_1_, *p*_2 _and *p*_3 _[5]. For the same reason and for simplicity, we implement our simulation algorithms only for the glucose regulation component. The relevant equations are  and .

Since we are only investigating the glucose regulation here, and will not estimate for the parameters involved in insulin secretion, we can for simplicity use a constant time scale *t*_0 _instead of *n*. A natural choice would be *t*_0 _= 60 min, so that  has the meaning of time in terms of hours, and *τ *∈ [0 3] during typical FSIVTT. The dimensionless parameters are then _1 _= *p*_1_*t*_0_, _2 _= *p*_2_*t*_0_, _3 _= *p*_3_(*I*_0 _- *I*_*b*_), and . The state variables are  and  = *Xt*_0_, with initial conditions (0) = 1 and (0) = 0. The program takes experimental insulin and glucose profiles *G*(*t*) and *I*(*t*) as inputs, estimates for [*p*_1 _*p*_2 _*p*_3 _*G*_0_] in the dimensional implementation; or calculates (*τ*) and (*τ*), and fits for [_1 __2 __3 __1_] in the dimensionless implementation.

**Figure 1:**
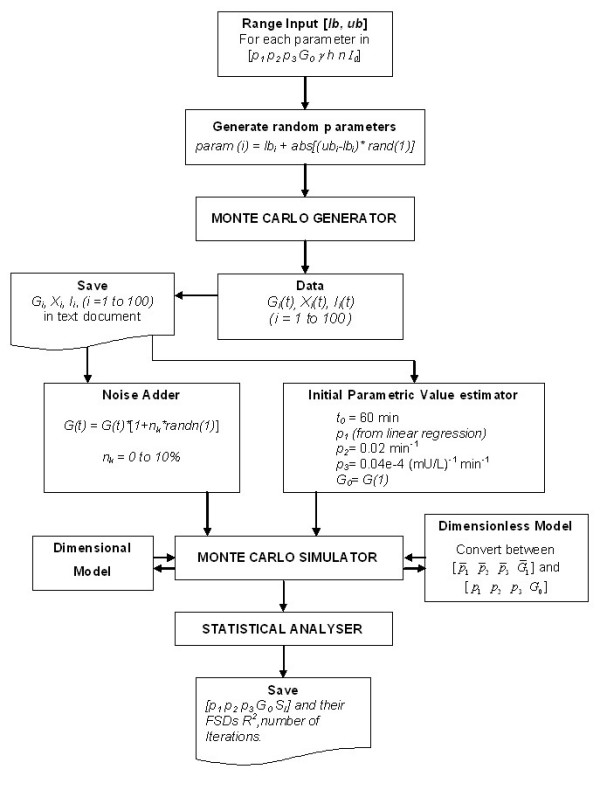
Flowchart of the Monte Carlo simulation.

### Monte Carlo simulation

The program flow chart for the Monte Carlo simulation is given in figure 1. Briefly, we first generated 100 simulated glucose and insulin temporal profiles using equation 1A–1C of MINMOD, with parameters randomly distributed within the following ranges: *p*_1 _∈ [1 4]·10^-2 ^min^-1^, *p*_2 _∈ [1.5 2]·10^-2 ^min^-1^, *p*_*s *_∈ [0.32 1.6]·10^-5 ^min^-2 ^(mU/l)^-1^, *n *∈ [1 4]·10^-1 ^min^-1^, *γ *∈ [1 4]·10^-3 ^(μU/ml) min^-2^, *h *∈ [92 125] mg/dl, *G*_0 _∈ [210 310] mg/dl, and *I*_0 _∈ [100 125] μU/ml. The basal levels of glucose and insulin were fixed at *G*_*b *_= 90 mg/dl, and *I*_*b *_= 14 μU/ml. We then used the generated profiles to fit for the parameters [*p*_1 _*p*_2 _*p*_3 _*G*_0_] in the dimensional formulation, and for [_1 __2 __3 __1_] in the dimensionless formulation. The fitting stringencies were set at ['TolFun', 1e-4, 'ToIX', 1e-4; 'MaxFunEvals', 1e6]. More details of these options of the Isqnonlin function can be found from Matlab's web site [22]. By default we adopted the Levenberg-Marquardt least-square minimization algorithm, though we have also tried the Gauss-Newton method for error minimization and obtained similar results.

To assess the robustness of our algorithm against noise we have also added Gaussian noise at the levels of 1–10% to the glucose profile of these 100 data sets, and examined the ability of our program to recover the true values of the parameters.

### Human FSIVGTT data

FSIVGTT data of 20 human subjects were kindly provided to us by the FUSION (Finland-United States Investigation Of Non-insulin-dependent diabetes mellitus) study group [23,24]. In this study healthy non-diabetic offspring of T2DM patients were recruited, and the tolbutamide modified FSIVGTT protocol [20] was administered to the individuals. Glucose and insulin were measured at 14 time points: 0, 2, 4, 8, 19, 22, 25, 30, 40, 50, 70, 100, 120, and 180 min. The initial values of the parameters were determined using the same method as described in the Monte Carlo simulation flowchart in figure 1.

## Results

### The dimensionless physiological indices in MINMOD

#### Choice a

In this formulation, the insulin sensitivity index becomes . The new definition gives a value that is the original *S*_*I *_times the total basal insulin secretion during the time scale of the glucose facilitated glucose uptake. The new index naturally includes both insulin sensitivity, and a measure of insulin secretion.

The Glucose effectiveness becomes . *p*_1 _represents the dissipation rate of glucose without insulin regulation. It is natural that in its own scale it becomes of unit value.

The first phase pancreatic responsiveness is . Notice that it can be also written as , where  is the total endogenous insulin secretion during the first phase response, and  represents the total glucose uptake due to glucose facilitated, insulin-independent mechanism. Therefore  represents their ratio normalized by their baseline levels. The second phase pancreatic responsiveness is .

In the new formulation, all rate constants are normalized with the time scale of glucose facilitated glucose uptake, and measures of insulin secretion are normalized with respect to glucose uptake.

#### Choice b

The insulin sensitivity index is now . In this formulation the new index depends on both insulin sensitivity *S*_*I*_, and total basal insulin secretion during the first phase, which reflects pancreatic function. It is reminiscent of Dl. Two critical components are involved in the regulation of plasma glucose: the pancreatic responsiveness (insulin release responding to changes in plasma glucose), and the efficiency of insulin facilitated glucose uptake (insulin sensitivity).  naturally includes both in its definition. Like Dl it has the potential to better characterize an individual's ability to regulate glucose than a measure of either component alone.

The glucose effectiveness becomes . The new index has the meaning of relative effectiveness of glucose facilitated glucose disappearance and insulin facilitated insulin disappearance.

The first phase pancreatic responsiveness  is now the ratio between the variation in insulin and the variation in glucose during FSIVGTT. The second phase response is . It reflects the rate of second phase insulin secretion rise per unit glucose stimulus, normalized by insulin's own disappearance rate and the baseline levels of insulin and glucose.

#### Choice c

The dimensionless counterpart of the insulin sensitivity index is now . Thus the disposition index Dl, which has been found to be predictive of disease pathology [6,9], is naturally defined by the system.

The glucose effectiveness becomes , which reflects the relative effectiveness of glucose facilitated glucose disappearance and insulin facilitated insulin disappearance.

The first phase pancreatic responsiveness becomes . This result implies that Dl can be interpreted as the insulin sensitivity at unit first phase insulin response. The second phase responsiveness is . The new index reflects the rate of second phase insulin secretion rise per unit glucose stimulus, normalized by insulin's own disappearance rate and the variation ranges of insulin and glucose.

This is by far the most natural choice to non-dimensionalize MINMOD. It normalizes the variation in glucose and insulin. Out of the three choices, it leads to the most simplified expressions of model equations, initial conditions and steady state solution. Importantly, the new formulation directly leads to the definition of Dl, and points out that it is an important index for the dynamic regulation of glucose.

### Comparison of the Monte Carlo simulations

#### Simulation performance

We have found that the simulation in dimensionless formulation is able to converge 20% faster, with mean number of iterations 6.4 ± 2.7 (Dim'less) versus 8.0 ± 1.4 (Dim'nl), p < 0.001 before converging. On average, each iteration in the dimensionless program also takes ~20% less time than one iteration in the dimensional program. On an Dell OptiPlex GX620 PC with Pentium^® ^dual 3 GHz CPU and 2 GB of RAM, the average CPU times are 0.122 s/per iteration in the dimensionless implementation versus a 0.159 s/per iteration in the dimensional implementation. Therefore the dimensionless implementation takes ~50% less simulation time.

**Figure 2:**
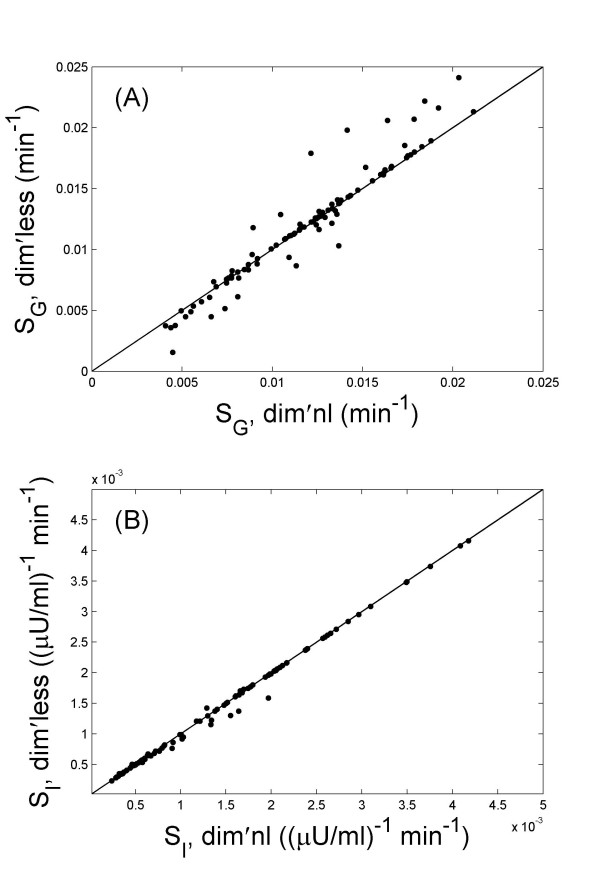
The comparison of parameter estimations for *S*_*G *_(A) and *S*_*I *_(B) between the simulations using the dimensional (dim'nl) and the dimensionless (dim'less) formulation of MINMOD. The results are from the 100 simulated data sets. Good agreements are observed in both cases.

The parameter estimation from the two programs agree well, with correlation coefficients 0.96, 0.98, 0.97, and >0.99 for the estimated *p*_1_, *p*_2 _*p*_3 _and *G*_0 _values, respectively. Figure 2 presents the estimated values of *S*_*G *_and *S*_*I*_, from the two implementations for all 100 data sets.

The precision in parameter estimation, reflected by their fractional standard deviation (FSD, defined as the ratio of standard deviation of the parameter-estimate to the mean value of the parameter-estimate), and goodness of fit *R*^2 ^are not significantly different between the two implementations. The results are summarized in table 2. Though the estimations of *p*_2 _and *p*_3 _seemed to be significantly off from their true values, the estimations of *S*_*I *_is much better in both dimensional and dimensionless implementations.

**Table 2:** Comparison between the dimensional and the dimensionless implementation of MINMOD. Results are from 100 Monte Carlo simulations.

Comparisons	Dimensional	Dimensionless	Significance
Number of iterations	8.0 ± 1.4	6.4 ± 2.7	P < 0.001
*R*^2^	0.97 ± 0.02	0.97 ± 0.03	N.S
*p*_1 _% difference from true value	(53.2 ± 7.6)%	(53.2 ± 9.6)%	N.S.
*p*_1_, FSD of estimation	(14.1 ± 2.7)%	(14.9 ± 6.0)%	N.S
*p*_2_, % difference from true value	(188 ± 160)%	(187 ± 155)%	N.S.
*p*_2_, FSD of estimation	(11.6 ± 1.6)%	(12.1 ± 3.5)%	N.S
*p*_3_, % difference from true value	(513 ± 324)%	(505 ± 317)%	N.S.
*p*_3_, FSD of estimation	(11.6 ± 1.7)%	(12.1 ± 3.5)%	N.S
*G*_0_, % difference from true value	(1.3 ± 0.4)%	(1.3 ± 0.4)%	N.S.
*G*_0_, FSD of estimation	(0.1 ± 0.04)%	(0.1 ± 0.06)%	N.S
*S*_*I*_, % difference from true value	(52.4 ± 25.5)%	(52.1 ± 25.5)%	N.S

#### Robustness against noise

**Figure 3:**
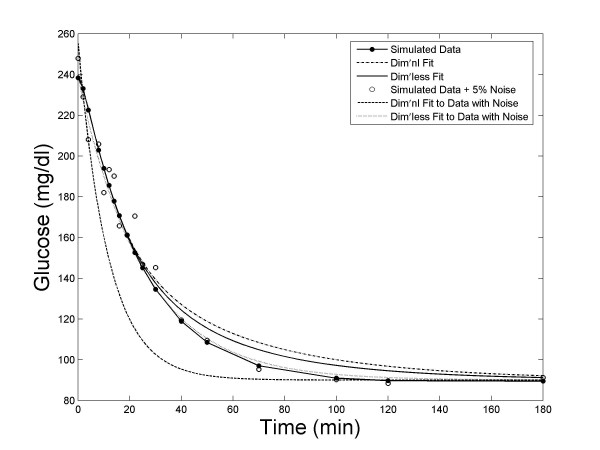
An example of model fitting to the Monte Carlo simulation data. The fitting algorithm using the dimensionless formulation of MINMOD is able to achieve better fit to data, and is less sensitive to noise.

**Figure 4:**
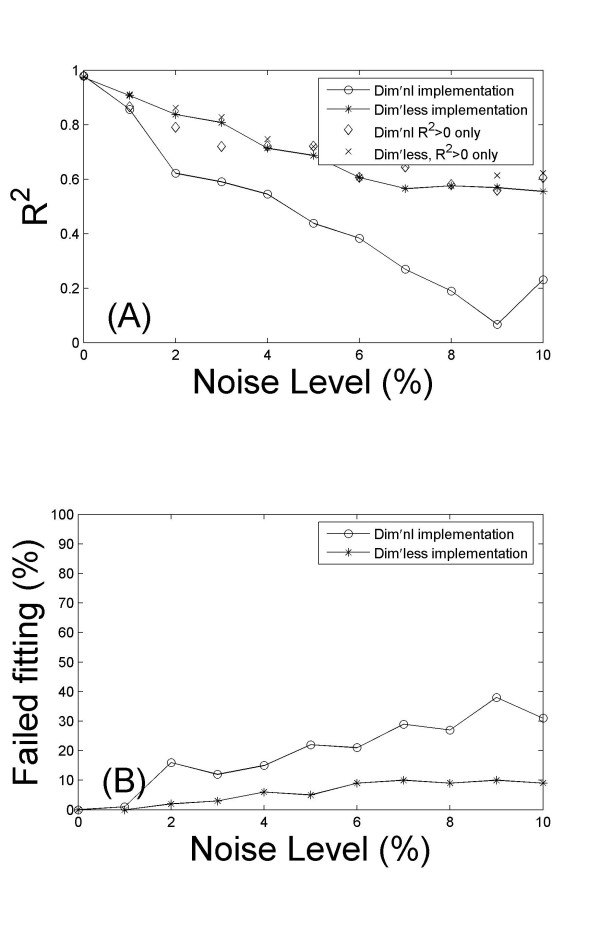
Comparison of the robustness against noise. Data shown are the results from 100 Monte Carlo simulations, with 0–10% noise added. (A) The average goodness-of-fit *R*^2 ^is plotted against the noise level. Overall the dimensionless implementation is able to achieve better model fitting at all noise levels. (B) The number of failed fitting (defined to be those with *R*^2 ^< 0) is more in the dimensional implementation than the dimensionless counterpart at all noise levels.

We have found that the dimensionless implementation of MINMOD is significantly more robust against noise. Figure 3 presents the fitting by the two methods to one of the Monte Carlo glucose profiles, and to the same profile after 5% noise was added. It shows that the simulation using the dimensionless formulation of MINMOD was less sensitive to noise. When we examined the statistics from all 100 data sets, we found that this is a general feature. Figure 4A presents the mean *R*^2 ^of fitting, as a function of the noise level. A better performance of the dimensionless implementation is evident.

When there is noise in the data, we observed that the program failed to converge in parameter estimation for some datasets within the default tolerance. The number of failed fitting goes up with increasing amount of noise. In real clinical/laboratory set up, noise in measurements are unavoidable, and it may have been a major factor that contributed to failure of parameter fitting some investigators have experienced with MINMOD [25,26]. Using a threshold of *R*^2 ^> 0, the failure rate is plotted against the noise level in figure 4B. Clearly, the simulation using the dimensionless formulation of MINMOD is significantly more robust. We have also calculated the mean *R*^2 ^after removing the failed fittings. The performance of dimensionless method is still better (figure 4A).

**Figure 5:**
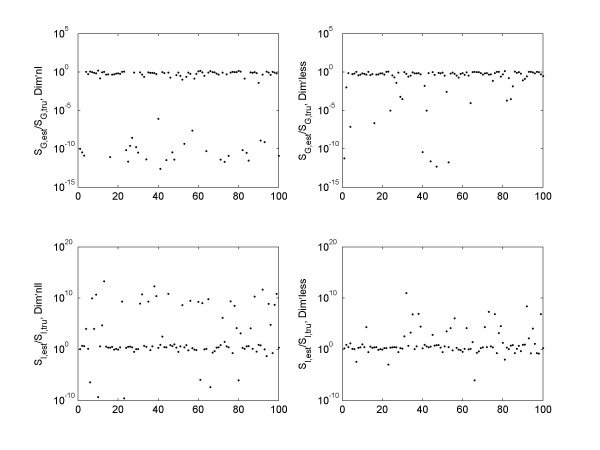
The ratio between estimated and true values of *S*_*G *_(top panels) and *S*_*I *_(bottom panels) is compared between the dimensional (left panels) and dimensionless implementation (right panels), for the 100 data sets with 1% noise.

**Figure 6:**
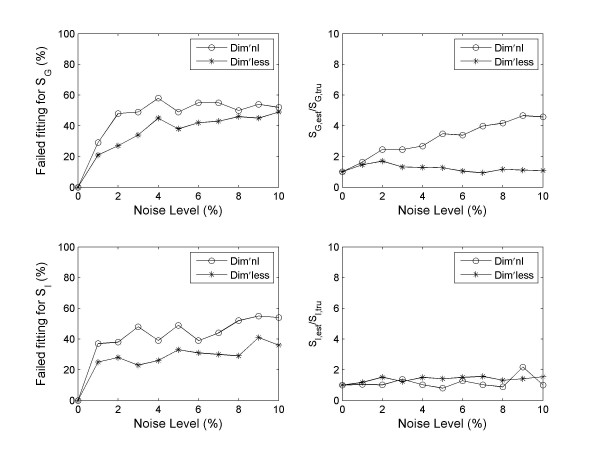
Comparison of the robustness in parameter estimation. The right panels show the number of failed estimations (defined by > 5 fold difference with the true values) for *S*_*G *_and *S*_*I *_as functions of noise level. The dimensionless implementation is better at recovering the true values of the parameters. The right panels plot the ratio of the estimated to true parameter values for those after removing failed fittings. The data points at 0% noise was normalized to one.

When there is noise, the dimensionless implementation is also able to better recover the true values of the parameters. In figure 5 we plot the ratio between estimated values of *S*_*G *_(top panels), *S*_*I *_(bottom panels) and their true values (the values used to generate the simulated data), at 1% noise level. Both methods were able to obtain reasonable good parameter estimation for majority of the data sets. However, there are some data sets where the estimated parameters were orders of magnitudes away from the true values. Using a 5 fold difference from true value as a cut off value, the number of failed parameter estimations is plotted against the noise level in figure 6. Overall the dimensionless implementation is able to better recover the true value (p < 0.001 for both *S*_*G *_and *S*_*I*_). It is worth pointing out that the data sets that failed in parameter estimations do not overlap completely with those that failed to reach a positive *R*^2^. There are some data sets which upon the addition of noise led to erroneous parameter estimations, but the fitting was reasonable with *R*^2 ^> 0.8.

After removing data sets with erroneous parameter estimations, we have calculated the mean ratio of the estimated *S*_*G *_and *S*_*I *_to their true values, and plotted their values against noise levels in the right panels of figure 6 (normalized by the first data points). The estimation in *S*_*G *_is more robust with the dimensionless method, whilst no significant difference was observed for the *S*_*I *_estimation.

### Human FSIVGTT data

Overall it takes many more iterations to fit the human FSIVGTT data than the simulated data. The improvement in speed with the dimensionless implementation is more significant in human data: 14.8 ± 8.8 iterations versus 36.3 ± 74.8 iterations, a 2.5 fold reduction with p = 0.02. Interestingly, the gain in CPU time per iteration is also more significant. Using the same PC, it is 0.115 s versus 0.210 s per iteration, leading to an almost 5 fold gain in CPU time.

**Figure 7:**
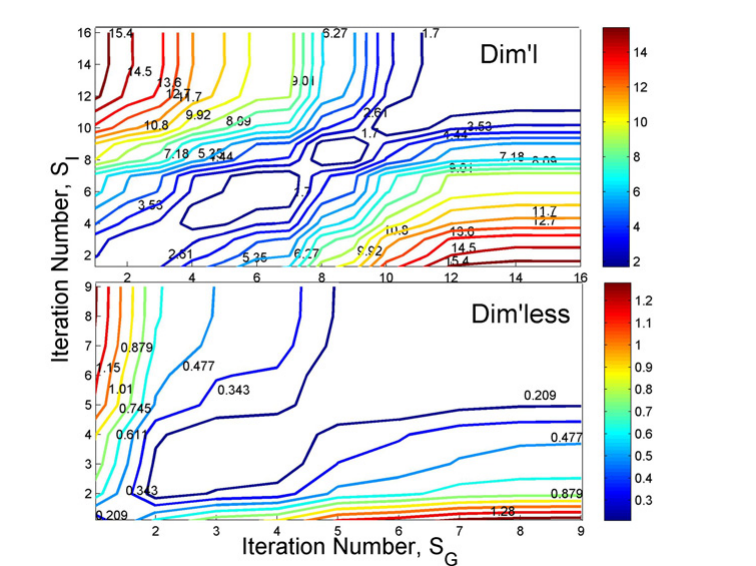
Contour plot of convergence in error minimization versus the number of iterations, for subject 9 in the FUSION data set. The levels of the cost-function in the contour plot are represented through their color, and is labeled on each contour. Top: Simulation using the dimensional formulation. Bottom: Simulation using the dimensionless formulation.

Figure 7 compares the contour plot of convergence between the two methods for subject 9. Contour plot of a non-linear least square minimization problem can help understand the nature of convergence, the magnitude of error and the number of iterations for both dimensional and dimensionless models implemented. In it the contours represent the values of the cost function (in this case, the norm of the error between the experimental and predicted glucose profiles) after each minimization iteration. As we can see from the figure, starting from the iteration 1, the cost function slowly decreases its magnitude and changes its color from "red" to "blue". The simulation using the dimensionless formulation of MINMOD exhibits much faster convergence to the cost-function minimization than the dimensional formulation.

**Figure 8:**
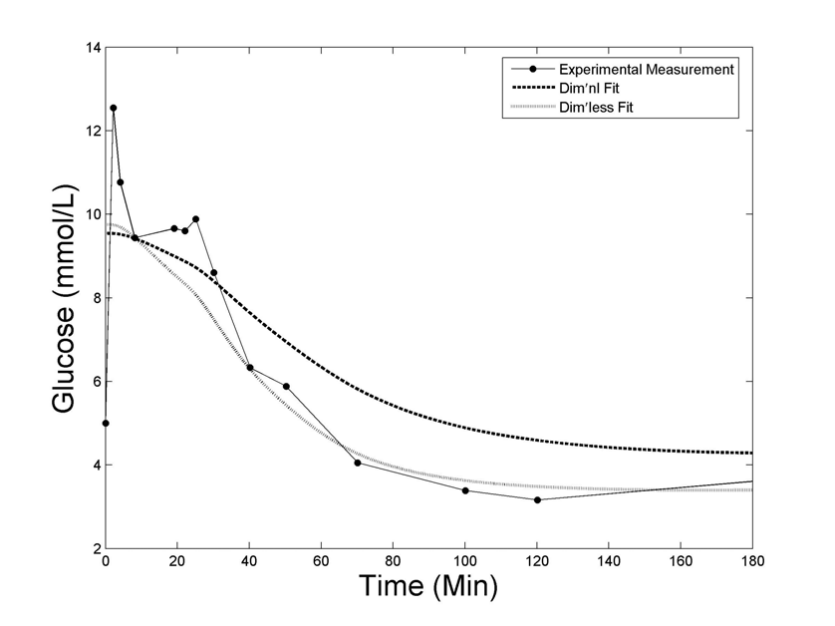
An example (subject 2) of the glucose measurements of the FUSION FSIVGTT data, and the model fittings using both dimensional and dimensionless formulation of MINMOD.

Out of the 20 subjects, dimensional implementation failed to reach a fit (*R*^2 ^< 0) for one individual (subject 2), whilst the dimensionless implementation was able to reach a good fit with *R*^2 ^= 0.93. We have played with different initial conditions to see that if the fitting by the dimensional program can be salvaged. With 4 different sets of initial parameter values, only one led to positive fitting with *R*^2 ^= 0.84, a value that is still significantly lower than what can be achieved with the dimensionless program. Figure 8 shows the fitting results for this subject when both methods reached a positive fitting. A close examination of its glucose profile indicated that the measurements were most likely compromised with noise. From 8 to 25 min the glucose rose unexpectedly while it should drop monotonically. This result is consistent with our findings in the Monte Carlo simulation where the dimensional implementation is found to be more sensitive to noise.

**Figure 9:**
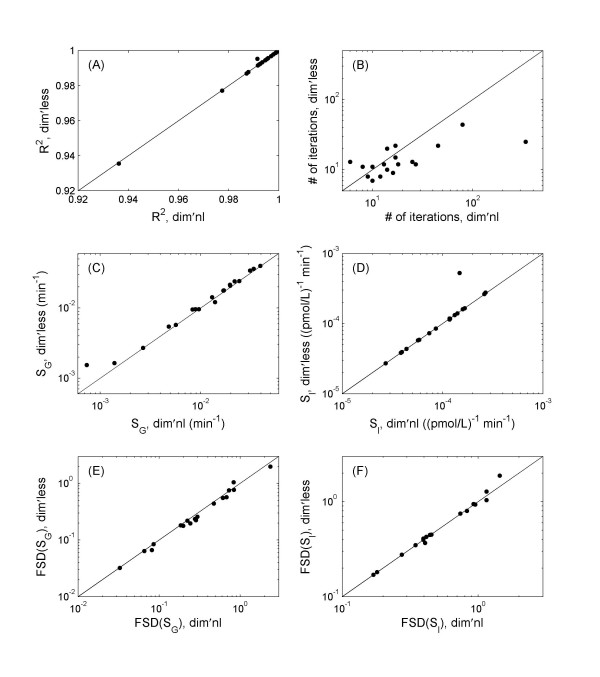
Comparison of the model fitting between the two methods for the FUSION data sets. (A) *R*^2^. (B) Number of iterations it takes to reach a fit. (C) Estimated *S*_*G *_values. (D) Estimated *S*_*I *_values. (D-E) FSD in parameter estimation of *S*_*G *_and *S*_*I*_.

Excluding subject 2, Figure 9 presents the results for the remaining 19 individuals using both dimensional and dimensionless implementation, including the goodness of fit *R*^2^, the number of iterations before reaching the fit, the estimated *S*_*G *_and *S*_*I*_, and the FSD of the parameter estimations. All data sets can achieve high *R*^2^, 0.991 ± 0.014 (Dim'less) versus 0.991 ± 0.015 (Dim'nl).

The estimations in *S*_*G *_and *S*_*I *_by the two approaches correlate highly (figures 9C–9D), with correlation coefficient 0.991 and 0.999 respectively. There is a consistent small difference in *S*_*G *_estimation, with the values derived using the dimensionless implementation ~11.9% higher on average than the dimensional implementation (p = 0.002). There is no difference in *S*_*I *_estimation (p = 0.33). The FSD in the parameter estimation also correlate well (figures 9E–9F). The FSD in *S*_*G *_estimation in the dimensionless implementation exhibits a significant (p = 0.007) albeit small improvement over the dimensional counterpart, suggesting that the higher *S*_*G *_value derived by the dimensionless implementation could be more accurate. The FSD in *S*_*I *_is not different (p = 0.26). All p values were obtained using the paired t-test on the logarithm transformed data, as the distribution of the transformed data is much less skewed (which can be seen from figure 9).

## Discussion

In diabetes research and clinical practice, it is very important to assess β cell function and insulin sensitivity, so as to evaluate the pathological status and risk of an individual. Many tests have been designed, and numerous indices have been defined. Using insulin sensitivity as an example, over a dozen have been experimented [27,28], none has been deemed best at predicting disease risk. These indices were all associated with certain units by definition. Some (Cederholm index [29] for example) would include several constants in the definition to accommodate the conversion between different units in measuring glucose or insulin concentration. Some indices are very similar by nature, like the Gutt index [30] and the Cederholm index [29], but can appear to be quite different as the same quantities were defined in different units. Therefore, when using these indices one must follow exactly their proposed forms and the glucose/insulin concentration units in order to obtain meaningful results. These make the utilization of and the comparison between different indices extremely clumsy. A dimensional analysis and definition of dimensionless parameters will eliminate most of the problems. In addition, it has been found that a combination of several measures could predict disease risk better than individual ones [31]. However, it is not clear how many measures are needed for best prediction.

As we have stated in the background section, a dimensional analysis can help us to further understand the model structure, identify the degree of freedom in the model and a non-redundant set of dimensionless parameters that together determines the dynamics of system and its sensitivity to pathological changes. Specifically, our analysis of MINMOD revealed that it has seven DOF, hence a full description of the metabolic control of glucose tolerance requires seven independent dimensionless parameters. It further offers a candidate set for the seven free parameters, which includes , and _1_. In the original analysis of FSIVGTT by dimensional MINMOD [1,2,5], four metabolic indices (*S*_*I*_, *S*_*G*_, *φ*_1 _and *φ*_2_) were defined that utilized only three of the seven DOF (_1_, , and ). Four additional DOF remain to be explored: , _1_, _1_, and a combination of _2 _and _3 _that is independent of  ( for example). These additional indices together with the ones that have already been utilized can potentially offer a better, more complete description of the metabolic control of glucose in humans, as well as its dysfunctions under pathological conditions. We hypothesize that together the complete set of seven DOF could lead to more accurate disease risk prediction.

Existing evidence from clinical reports have indicated the relevance of the additional four indices. _1 _or _1 _each by itself could be a meaningful index, as they each reflect the basal glucose or insulin level with respect their dynamic range of change. An alternative index of glucose effectiveness that has been in use is [32], where *D *is the glucose bolus dose (mg/kg), AUC is the area-under-curve of the glucose concentration excursion above basal. _1 _is related to it by , where  is the ratio between total basal glucose uptake and the glucose bolus dose. The product of _1 _and _1 _is also of interest, as , where *HOMA *= *G*_*b*_·*I*_*b *_is the HOmeostasis Model Assessment of the steady state β cell function and insulin sensitivity [33]. Recently, a new composite index for insulin sensitivity defined using both  and *p*_2 _was investigated [34]. Therefore it would be of interest to explore the physiological meaning of the remaining 4 DOF further, and their potential as disease markers.

The fitting failure rate in the Monte Carlo simulation is high. This is expected to improve if we implement more sophisticated initial parameter value estimation and baseline correction algorithms, as found in [5]. In addition, the model fitting to the 20 FUSION subjects seems to be much better than the fitting of the Monte Carlo data sets at any noise levels, with higher *R*^2 ^values, less failure rate and better agreements between the two methods. This is likely due to the fact that the FUSION used the modified FSIVGTT protocol, which is known to lead to better parameter estimation. As insulin secretion mechanism in modified protocol is not known, it is not possible to run a Monte Carlo simulation for the modified protocol.

## Conclusion

In this work we performed dimensional analysis of MINMOD. We found that with a non-dimensionalization choice that normalizes the variation in glucose and insulin during FSIVGTT, the pathologically important Dl index is naturally defined in the model and it has the meaning of the insulin sensitivity at unit first phase pancreas response. Several additional dimensionless indices were also defined, which potentially offer means to better characterize the metabolic control of glucose and its dysregulation under pathological conditions. In addition, we have explored the advantages of computer simulation of MINMOD using its dimensionless formulation. Using simulated data as well as real human FSIVGTT data, we found that whilst the new approach gives highly correlative (correlation coefficients all above 0.96) parameter estimations to the original dimensional formulation, it led to significantly improved simulation speed and is much more robust against noise.

## Authors' contributions

XW drafted the initial study design. AP and XW performed the mathematical analysis, coding and the numerical simulation, and drafted the manuscript. SG and RB contributed to the discussion and the interpretation of results. All authors shared discussions and participated in finalizing study designs and in manuscript writing. All authors read and approved the final manuscript.
